# Evaluating the Appropriateness of Selected Foundry Sands for the Casting of Reactor Housings: A Study Based on Physicochemical Characterization Outcomes

**DOI:** 10.3390/ma17246068

**Published:** 2024-12-12

**Authors:** Paweł Gara, Ewa Wisła-Walsh, Tomasz Bajda

**Affiliations:** 1 Faculty of Mechanical Engineering and Robotics, AGH University of Krakow, al. A. Mickiewicza 30, 30-059 Krakow, Poland; pgara@agh.edu.pl (P.G.); wisla@agh.edu.pl (E.W.-W.); 2 Faculty of Geology, Geophysics and Environmental Protection, AGH University of Krakow, al. A. Mickiewicza 30, 30-059 Krakow, Poland

**Keywords:** spheroidization, physicochemical properties, thermal analysis, quartz sands

## Abstract

In the case of desulfurization and spheroization of cast iron using the in-mold method, in which the treated cast iron is poured into the reaction chamber and placed in the casting mold, the mineral raw material of the mold should support these processes. Therefore, it is important to know the physicochemical properties of the materials selected for the production of casting molds and to learn about the phenomena occurring during their pouring. The research presented in this paper was carried out on quartz, magnesite, chromite, and olivine sands. The results not only provide a comprehensive understanding of these materials but also have significant implications for reactor housing casting. Two of the three tested quartz sands meet all the standards, allowing quartz raw materials to be foundry sands. Marked by the authors of this work, P11 sand, which is classified as 1K grade by the seller, does not meet the requirements of the Polish standard PN-85/H-11001 for this grade and should be classified as 2K grade. At the same time, attention was drawn to relatively considerable weight losses at 1350 °C for the tested quartz raw materials. More significant losses on ignition were found for magnesite sand than the value permitted by the Polish standard, which should be associated with the fact that derivatographic tests were carried out in an oxidizing atmosphere. In the analysis made for olivine sand, the obtained data indicated that the magnesium content is slightly below the requirements of the relevant standard; on the contrary, the iron content exceeds the standard requirements. Analytical data obtained for chromite sand indicated that it meets the PN-91/H-11007 standard regarding chemical composition, but X-ray diffraction tests showed that the tested sample is not chromite but magnesiochromite. The results of grain size distribution, chemical composition, X-ray diffraction, SEM/EDS, and TG/TG presented in this paper show that before starting the production of a specific molding mixture, each time most of the parameters characterizing sand used should be controlled because the properties may differ from the manufacturer’s declaration.

## 1. Introduction

The primary product of cast iron smelting in cupolas is gray cast iron, which does not require additional processes before, during, or after smelting [1,2,3]. However, for cast irons with enhanced mechanical properties, like vermicular and nodular cast iron, the transformation of flake graphite into spheroidal graphite is necessary [4,5]. The spheroidization process is efficient when the sulfur content in cast iron is approximately 0.02%, requiring sulfur reduction before spheroidization [6].

In current methods, molten cast iron is treated for desulfurization and spheroidization outside the casting mold, and then the refined cast iron is poured into molds [7,8]. These processes are typically conducted in a casting ladle using the “wire” method or with specific additives, followed by pouring the cast iron into the molds [9,10,11,12]. The time between metallurgical treatment and casting can range from several to over ten minutes, during which the metal settles and reaction products separate from the bath.

In contrast, the in-mold method [13,14] involves spheroidization or vermicularization in a reaction chamber inside the mold. All metallurgical reactions occur when the liquid metal is poured into the mold, with processes lasting from about 8 to 20 s. These rapid reactions occur not only in the reactor but also within the gating system and mold cavity, meaning the mold material can influence the process [15,16]. Consequently, the properties of foundry sands used in molding support these reactions.

The term “sands” in the foundry industry (in analogy to mineralogy) denotes a number of natural (mineral) and synthetic materials with a grain size of 0.05 to 2.0 mm, which meet certain strength and thermal criteria at high temperatures (up to about 1450 °C) [17,18,19,20]. Natural sands include quartz, olivine, chromite, and zircon, while synthetic options include magnesite and corundum [17,19,21]. The sand selected must have the appropriate grain size, shape, chemical composition, and binder content depending on the casting size, material, and technique [22,23,24].

The following requirements are the chemical composition and the content of natural binders [25,26,27,28,29]. An equally important parameter that determines the possibility of using specific sand to make a mold is the value of the pH parameter, affecting the possibility of using a specific binder [17].

The most common molds used to manufacture iron alloy castings are quartz sands (SiO_2_) with a specific granulometric composition and acceptable amounts of contamination with chemical compounds that affect their fire resistance and pH value, mainly due to the price. Materials such as bentonite are used as the grain matrix binder, and in the case of chemically or thermally bonded sands, inorganic (e.g., hydrous sodium silicate) and organic (e.g., synthetic resins) binders are applied. The main technological requirement for molding sands obtained from these materials is having appropriate strength and filtration properties, determined by the permeability coefficient value, called permeability [17,29]. SiO_2_ (chemically quartz sand) forms two polymorphs: high-temperature α, which changes into the β form when cooled at 573 °C. The β-quartz is the main component of naturally occurring quartz sands. As the heating proceeds, α-quartz changes into tridymite (870 °C) and then into cristobalite, and at the temperature of 1470 °C, the process of sand melting and its glass transition begin. Polymorphic transformations of quartz accompanying its heating cause a decrease in its density and, consequently, an increase in the grain volume [30]. This is a disadvantageous phenomenon in foundry engineering and may cause the formation of defects such as “scabs”, “lines”, and “scars” [31,32]. The increase in grain volume is partially compensated for by shrinkage (up to about 15%) of the added hydrated binder (clay) [17]. Furthermore, quartz sands can transition into cristobalite at 1050 °C rather than tridymite, leading to varied thermal expansion [31].

If using silica sands as a matrix of molding sand is impossible, magnesite sands are used [16,17]. Magnesite sands are burnt-sintered (firing temperature from 1450 to 1850 °C) magnesites (magnesium carbonates), i.e., magnesium oxide (periclase) [16]. After firing, the obtained material is sieved to the specified particle size distribution. According to the Polish Standard PN-67/68-04 [28], Mg-P1 magnesite sand should contain at least 80% magnesium oxide, MgO, and Mg-P2 sand, and at least 72% MgO [28]. Additionally, when using magnesite sand in foundry engineering, it is required that the content of calcium oxide CaO must not exceed 3.5%, and the SiO_2_ content should not exceed 7% [28]. Binding materials (binders) for magnesia sands may be clay, water glass, ethyl silicate, or other neutral or alkaline materials. The typical binders for magnesite sands include water glass and other neutral or alkaline materials, as acid-hardened resins are incompatible [16].

Olivine sands are preferred for their superior thermal properties compared to quartz sands, although their high pH makes them less compatible with acid-hardened organic binders [22,23,24,25,26,27]. Olivine sands are most often obtained from olivine rich in forsterite, Mg_2_SiO_4_. In the composition of the olivine used in the foundry industry, the ratio of forsterite to fayalite (Fe_2_SiO_4_) is 9:1, MgO content is not lower than 49%, bound SiO_2_ content is not higher than 42%, and the content of impurities (Fe_2_O_3_ + Al_2_O_3_ + TiO_2_) is not higher than 1% [17]. The grains of olivine used in the foundry are sharp-edged, and the average grain size, dL, is 0.1–0.3 mm. The most common binders for olivine sands are bentonites, starch binders, isocyanate-hardened alkyd resins, water glass, and novolak-type resins [17,26,33].

Chromite sands, consisting of chrome spinels like FeCr_2_O_4_, are used for their low and uniform thermal expansion, high thermal conductivity, and resistance to metal oxides and slags [17]. Chromite sands must contain more than 44% Cr_2_O_3_ and maintain low levels of SiO_2_ to meet foundry standards [27]. However, their high pH and cost limit their use in some technologies. The average grain size for treated chromite sands, dL, is usually 0.2 to 0.25 mm, and their shape is usually angular. In Poland, the properties of chromite sands for applications in the foundry are regulated by the Polish Standard PN-91/H-11007 [27], according to which three types of chromite sands are distinguished in terms of graining. The pH value of chromite sand, as in the case of olivine sands, prevents many binders in molding sands, limiting its use in many technologies. Another limitation of the use of chromite sands is their high price.

Molding sand is used in the foundry industry to prepare molds during metal casting. Its properties improve the casting metal’s quality. When adequate sand is used, casting faults that may occur during the mold preparation and casting are considerably reduced.

This work aims to evaluate the appropriateness of selected foundry sands (quartz, magnesite, chromite, and olivine) for casting reactor housings, focusing on their physicochemical properties. The aim is to assess the compatibility of these sands with casting processes, such as desulfurization and spheroidization, by analyzing their chemical composition, grain size distribution, and thermal behavior. The results of this study will help determine whether the selected sands meet the required standards for foundry use and highlight any deviations from these standards, thereby guiding future foundry material selection.

In current industrial practices, molding materials are typically chosen based on manufacturer specifications, with limited verification of their actual properties. However, discrepancies between declared and actual sand properties, such as grain size distribution, chemical composition, and thermal behavior, can significantly impact their performance. This mismatch often leads to defects like slag inclusions, surface scabs, and dimensional instability in the final castings. Furthermore, the role of molding sand properties in supporting key metallurgical processes, such as sulfur reduction and graphite spheroidization, remains underexplored in the context of reactor housings.

This study addresses these knowledge gaps by systematically characterizing the physicochemical properties of the selected sands using advanced techniques, including XRD, SEM-EDS, and TG/DTG analysis. By correlating these properties with their performance in high-temperature applications, this research comprehensively explains their suitability for foundry use. The findings contribute to developing improved quality control practices and offer actionable insights for optimizing sand selection, ultimately enhancing casting efficiency and product quality.

## 2. Materials and Methods

In this study, the sands are classified using the Polish Standard PN-85/H-11001 [27] for silica sands and similar standards for non-silica sands. This classification includes the following terms:

- Grade: refers to the sand’s quality based on compliance with specific chemical and physical parameters, such as binder content, grain composition, and impurities.
- Group: denotes the granulometric characteristics of the sand, such as fine, medium, or coarse grains, determined through sieve analysis or laser diffraction methods.
- Class: represents the sand’s suitability for specific foundry applications, combining parameters like thermal stability, chemical purity, and mechanical strength.

### 2.1. Preparation of the Test Material

The physical and chemical properties of molding sands were tested for two samples of commercial quartz sands (sands P-3, P-12) and glass sand P-11. In the case of the latter sand, the preparation for testing consisted of sifting the fraction of the tested sand to meet the requirements for the grain size distribution of silica sands according to the Polish Standard PN-85/H-11001 [27]. The tested magnesite, chromite, and olivine sands are also commercial sands. Before starting the tests, the collected samples were dried at 110 °C for 3 h.

### 2.2. Grain Size Distribution

Measurements of the particle size and particle size distribution of the materials tested were carried out by the laser diffraction method on a Malvern Mastersizer 2000 (Malvern, Worcestershire, UK) apparatus. The measurements were performed in distilled water, with a refractive index of 1.33, as a dispersion liquid. The samples were treated with ultrasound at approximately the maximum power for 4 min. The ultrasound was derived from an ultrasound probe with a maximum power of 300 W. Malvern Mastersizer 2000 had a measurement uncertainty of ±1 μm for the reported particle sizes, with a detection range of 0.05 to 2000 μm. Mie theory was adopted to calculate the particle size.

### 2.3. Chemical Composition

The elemental composition of the investigated sands was determined by energy-dispersive X-ray fluorescence (XRF) using an Epsilon 3 Panalytical spectrometer (PANalytical, Almelo, Netherlands). The tests were conducted at a range of Na-Am on an apparatus equipped with a lamp with a 9W Rh, 50 kV, and 1 mA anode. The wavelength-dispersive X-ray fluorescence analysis had a detection limit of 0.01 wt.% for most major oxides. These uncertainties are based on calibration with certified reference materials (CRMs). XRF was utilized for elemental analysis due to its high accuracy for multi-element samples, particularly for foundry sands where chemical composition is critical. This is critical for assessing compliance with foundry standards (e.g., Polish standards PN-85/H-11001 [27] and PN-91/H-11007 [29]).

### 2.4. Phase Composition

The mineral composition of all materials was determined by X-ray diffraction (XRD) using X’pert (Panalytical, Malvern, UK) with a PW 3020 goniometer, CuKα radiation, and a graphite monochromator. The analysis was carried out within the angle range of 5–65° (2 Theta). XRD analysis was conducted with a measurement uncertainty of ±1% for phase identification. The instrument’s detection limit for minor phases was approximately 1% by weight, depending on the sample matrix. Panalytical X’PertHighscore software (High Score Plus v. 4.1) was used to process the diffraction data. The identification of mineral phases in the materials under study was based on the PDF-2 Release 2010 database formalized by the ICDD.

The morphological forms, microstructure, and chemical composition of the tested foundry sands were determined using a scanning electron microscope (SEM) FEI Quanta 200 FEG (FEI, Hillsboro, OR, USA) equipped with a system of chemical composition analysis based on energy-dispersive spectrometry (EDS) X-ray/EDS from the EDAX company (EDAX Inc., Mahwah, NJ, USA). SEM/EDS analysis examined the sands’ surface morphology, grain shape, and microstructural features. This method identifies surface irregularities affecting binding properties and permeability in molding sands. SEM-EDS also provides elemental distribution, which is critical for evaluating impurities such as Fe, Al, or Cr that can alter the sand’s refractory behavior.

The thermal analysis (TG/DTG/DTA) was performed on a Jupiter STA 449 F3 Netzsch coupled with a quadrupole mass spectrometer, Aeolos QMS 403C. Fifty milligrams of an air-dried sample were heated from 25 °C to 1350 °C in an alumina crucible at 30 °C per minute in flowing (40 m L^−1^ min^−1^) synthetic air. TG had a sensitivity of 0.01 mg, and the temperature measurement uncertainty was ±1 °C. Detection of phase transitions was accurate within ±2 °C. Thermal analysis is critical for understanding thermal stability, loss on ignition, and phase transitions in sands, especially during heating. These properties influence the sand’s thermal shock resistance and dimensional stability under casting conditions.

## 3. Results

### 3.1. Silica Sands

#### 3.1.1. Grain Size Distribution and Physicochemical Properties

**Figure 1:**
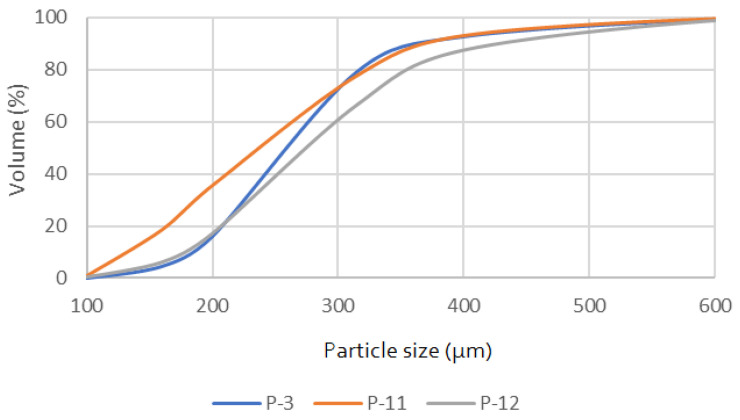
Particle size distribution for the tested quartz sands.

Figure 1 shows the particle size distribution for samples P-3, P-11, and P-12. The results suggest that P-12 has the most uniform particle size distribution among the three samples, with a higher concentration of particles around a specific size range. In contrast, P-3 and P-11 have broader distributions, with P-11 skewed towards smaller particle sizes.

Table 1 presents statistical data and classification of the sands by the Polish Standard PN-85/H-11001 [27].

The data in Table 1 show that in terms of particle size, the tested quartz sand samples meet the requirements of the Polish Standard PN-85/H-11001 [27]. However, P-11 sand was classified as 2K due to the homogeneity index (share of the most frequently represented fractions in the sieve analysis in relation to the total sample), which does not fit into the 80% required for the first category sands.

The data presented in Table 2 indicate that P-3 sand is practically pure silica. However, the content of the natural binder determined therein is inconsistent with the requirements of the Polish Standard PN-85/H-11001 [27]. The chemical analysis results (Table 3), in conjunction with their particle size distribution (Table 1), enable the classification of P-3 sand to the 4K grade.

As mentioned above, the high binder content disqualifies the sand from being included in the first three categories (for which the permissible binder content is 0.5%, 1%, and 1.5%, respectively). P-11 sand was classified as second-grade fine sand (2K) because it meets the requirements for this group of sands by the requirements of the Polish Standard PN-85/H-11001. In turn, sand P-12 meets all the requirements in the standard mentioned above. Therefore, it can be classified into the first group of quartz sands.

#### 3.1.2. XRD

**Figure 2:**
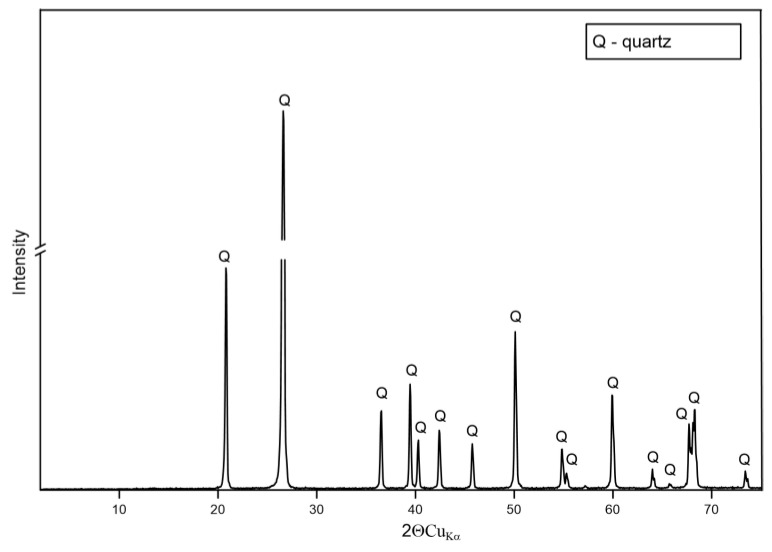
XRD pattern of sample P-3.

**Figure 3:**
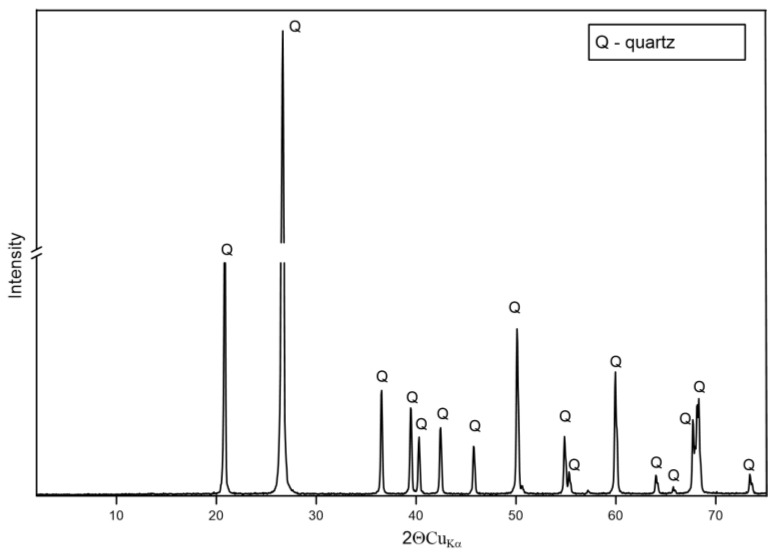
XRD pattern of sample P-11.

**Figure 4:**
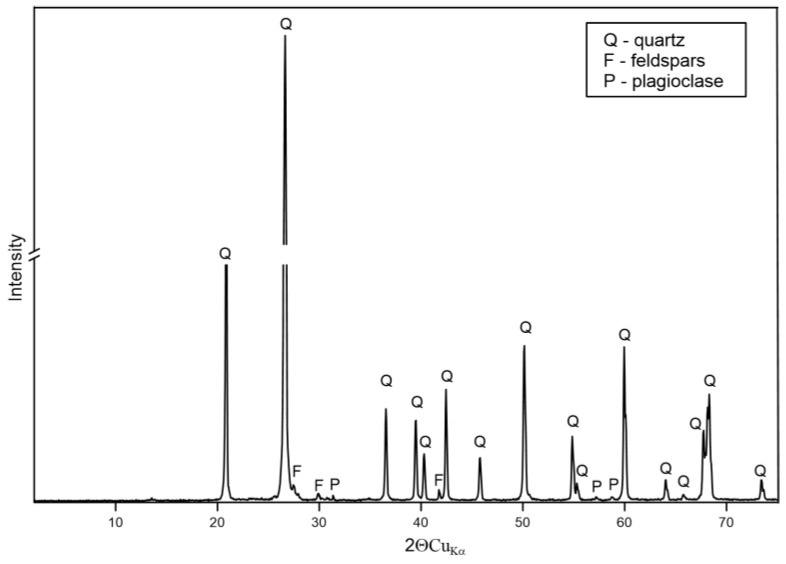
XRD pattern of sample P-12.

**Figure 5:**
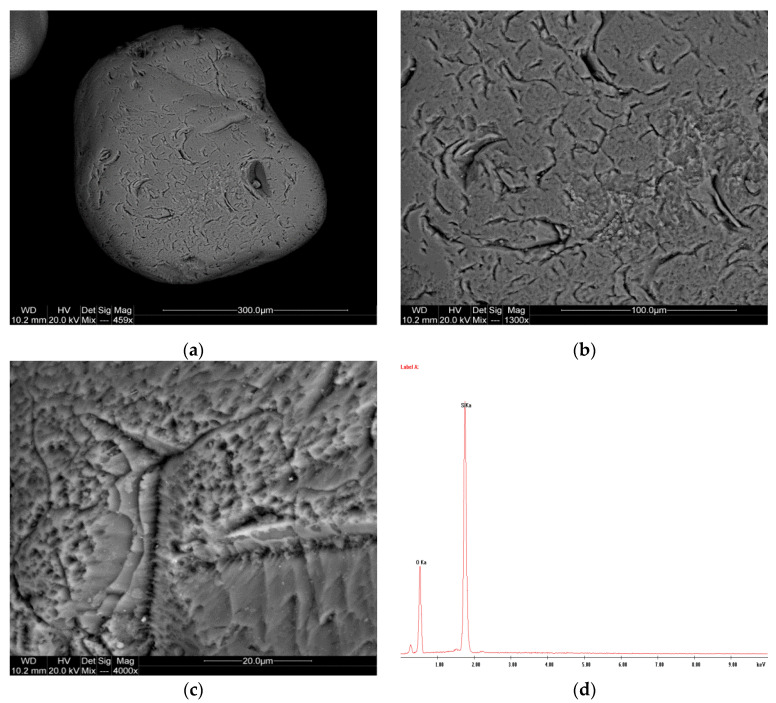
SEM images (**a**–**c**) and EDS spectrum (**d**) of the P-3 sample.

The results of the XRD analysis presented in Figure 2, Figure 3 and Figure 4 indicate that the major peaks observed in samples P-3 and P-11 are consistent with the typical reflections of quartz, indicating that quartz is the dominant mineral phase. The intensity and sharpness of the peaks suggest that the quartz in this sample is well crystallized. The XRD pattern of sample P-12 (Figure 5) is similar to those of P-3 and P-11, with prominent peaks corresponding to quartz. This sample also exhibits sharp, intense peaks, indicating high crystallinity. The consistency in peak positions with the other samples confirms that quartz is a major phase in P-12, with potential minor contributions from feldspars and plagioclase as indicated by any secondary peaks.

#### 3.1.3. SEM-EDS

**Figure 6:**
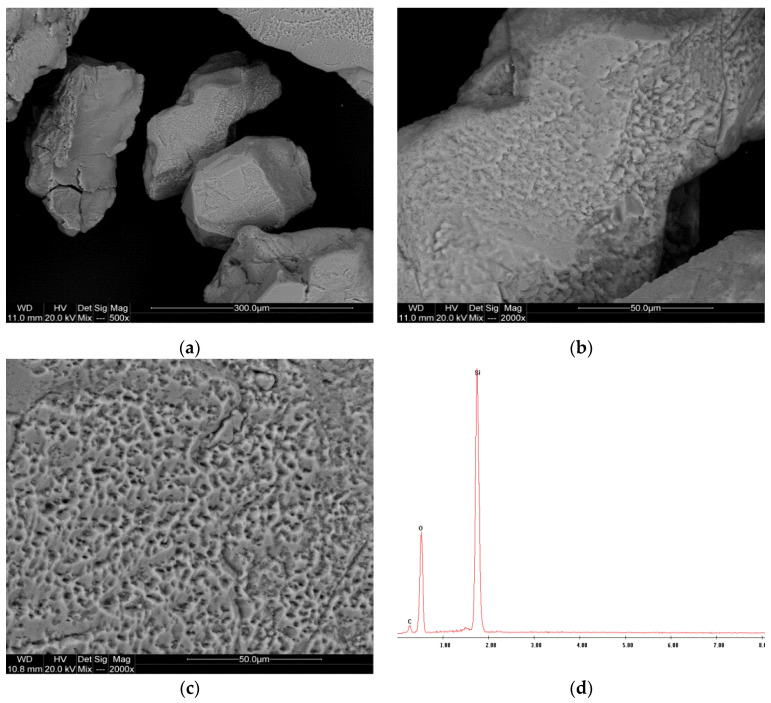
SEM images (**a**–**c**) and EDS spectrum (**d**) of the P-11 sample.

**Figure 7:**
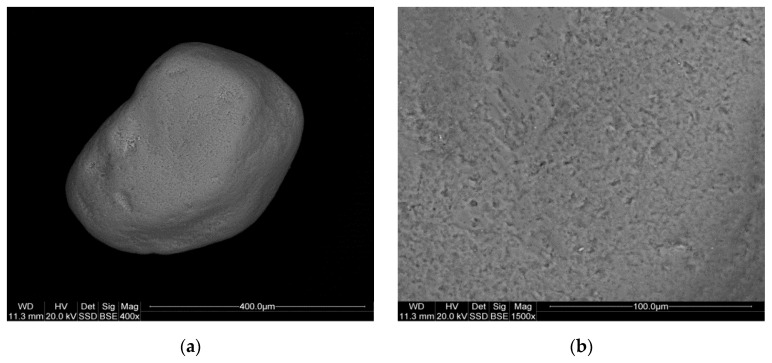

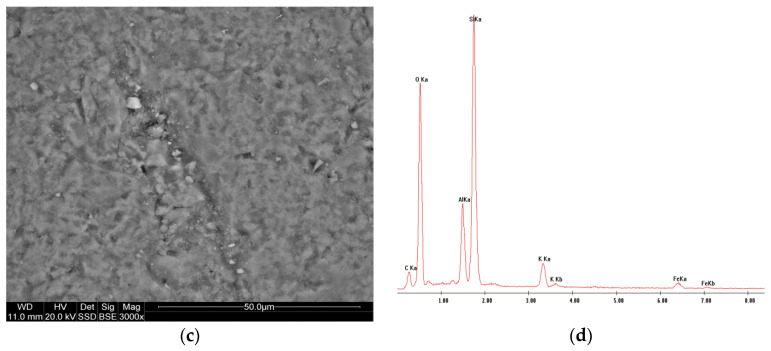
SEM images (**a**–**c**) and EDS spectrum (**d**) of the P-12 sample.

The analysis results for samples P-3, P-11, and P-12 are presented in Figure 5, Figure 6 and Figure 7.

The results of the SEM analyses of quartz sands presented above verified that most of the available sands in southern Poland are of river origin. Therefore, their grains were rounded during transport, and their surface was mechanically “grooved”. The SEM images of sample P-3 (Figure 5a) reveal a relatively uniform particle size with angular and irregular shapes typical of quartz sands. The surface texture (Figure 5b,c) appears slightly rough, which could indicate mechanical weathering processes. The particles exhibit sharp edges, suggesting minimal transportation. The EDS analysis shows (Figure 5d) a dominant peak for silicon (Si) and oxygen (O), consistent with the composition of quartz (SiO_2_).

The SEM images of sample P-11 (Figure 6a) also display quartz particles with angular shapes, but there is a noticeable difference in particle size distribution compared to P-3. The particles in P-11 seem more varied in size, with some larger grains visible. The surface texture appears smoother (Figure 6b,c) than in P-3, possibly due to different depositional environments. Similar to P-3, the EDS spectrum for P-11 shows strong peaks for silicon and oxygen (Figure 6d), confirming the quartz composition.

The image for sample P-12 (Figure 7a) indicates a more homogeneous particle size distribution, with particles generally smaller and more rounded than in P-3 and P-11. The particles’ roundness and smooth surface texture (Figure 7b,c) suggest that this sample has undergone more significant transportation or weathering processes. The EDS spectrum for P-12 (Figure 7d) shows Si and O as the predominant elements, consistent with quartz. The spectrum also reveals the presence of impurities (Figure 7d), with the presence of Al, K, and Fe, as confirmed by the presence of feldspars and plagioclase identified by XRD.

#### 3.1.4. TG/DTG

**Figure 8:**
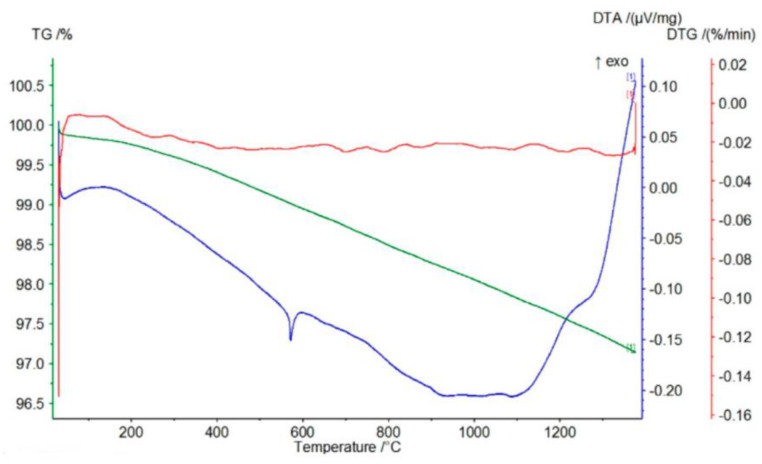
Thermal curves of sample P-3: TG curve (green), DTG curve (red), DTA curve (blue).

**Figure 9:**
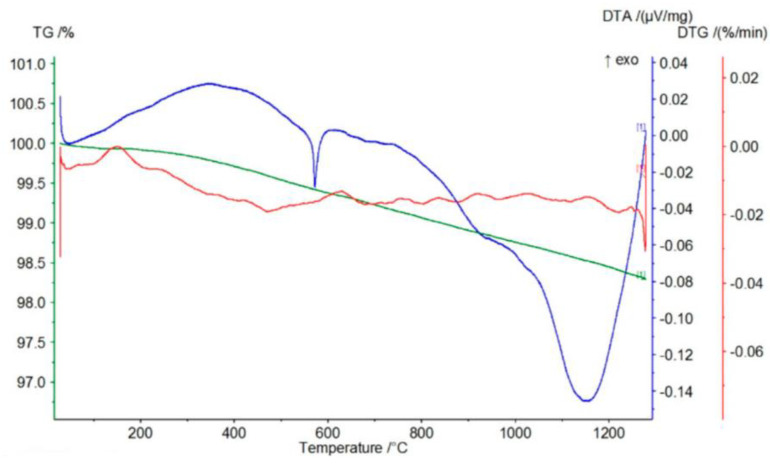
Thermal curves of sample P-11: TG curve (green), DTG curve (red), DTA curve (blue).

**Figure 10:**
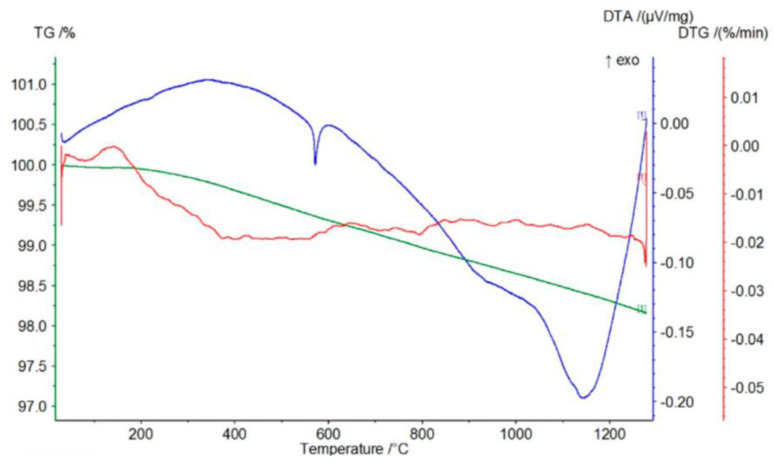
Thermal curves of sample P-12: TG curve (green), DTG curve (red), DTA curve (blue).

Figure 8, Figure 9 and Figure 10 represent the thermal effects during P-3, P-11, and P-12 analysis. An endothermic peak accompanies the TG-curve mass loss on the DTA curves at about 570 °C, which is responsible for the transformation of α-quartz into β-quartz. The DTA curves slope monotonically to about 1100 °C, passing through a slight maximum at about 1200 °C. A possible explanation for such a course of the DTA curve for the P-3 quartz sand is the direct transition of β-quartz into β-cristobalite [30,31]. The course of the TG/DTG/DTA curves presented in Figure 8, Figure 9 and Figure 10 and the data obtained on their basis, presented in Table 4, indicate that the P-11 and P-12 sands behave similarly to the P-3 sand, except that at a temperature of about 900 °C an inflection appears on the DTA curve rather than a pronounced peak.

The effect of the parameters of a given sand was studied at 1300 °C, revealing that deviations from standard requirements for sand grain size and composition lead to significant issues in mold performance. For instance, grains that do not conform to the required granulometric composition or exhibit excessive impurities may cause irregular thermal expansion. This inconsistency can create internal stresses within the mold, leading to cracking during the casting process. Furthermore, non-standard sand grains can contribute to surface defects in the final casting, such as scabs, scars, or inclusions. These defects compromise not only the aesthetic quality of the casting but also its mechanical properties and reliability, particularly in high-precision applications such as reactor housing production.

### 3.2. Non-Silica Sands

#### 3.2.1. Grain Size Distribution and Physicochemical Properties

**Figure 11:**
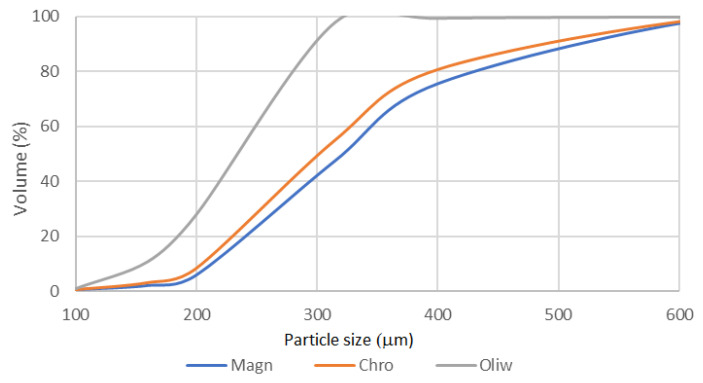
Particle size distribution for tested sands: P-4 magnesite, P-5 chromite, P-7 olivine.

Figure 11 presents the particle size distribution for samples P-4 (magnesite sand), P-5 (chromite sand), and P-7 (olivine sand). The magnesite sample exhibits a broad particle size distribution. Most particles fall within the 200 to 400 µm range, with a significant proportion of particles above 300 µm. This indicates that the magnesite sand is relatively coarse-grained compared to the other samples. The chromite sand shows a more narrowly distributed particle size, with most particles ranging between 100 and 300 µm. The olivine sand presents a distribution curve similar to that of chromite but with a slightly broader range. Most particles are distributed between 100 and 400 µm, indicating a slightly coarser grain size than chromite. Table 4 presents statistical data and classification of these sands by the Polish Standard PN-85/H-11001 [27].

Sieve analysis for magnesite sand shows that it meets the requirements of PN-85/H-11001) [27] regarding the grain size. Chromite sand was assigned to the grain group corresponding to coarse sand based on sieve analysis [27]. The results obtained for the sand P7-olivine allowed it to be classified into the grain group “fine” and grade 1K. The qualification was made by the Polish Standard PN-85/H-11001 [8] for quartz sands because there is no Polish standard for olivine sands.

The data in Table 5 reveal that the P-4 magnesite sand shows higher losses on ignition than the value allowed by the standard [27]. This should be related, on the one hand, to the storage method of the tested sand and, on the other hand, to the fact that derivatographic tests were carried out in the air.

In the case of chromite sand P-5, the above data indicate that during heating, there is no loss of mass but an increase, which is confirmed by TG and XRD analysis and which should be associated with the fact that the tested sand is not pure chromite but chromite with an admixture of magnesia chromite. In turn, the data in Table 6 indicate that the magnesium content in olivine (P-7) is only slightly lower than the requirements of the relevant standard [27]. On the contrary, the iron content quite significantly exceeds these requirements.

The measured values of the pH parameter for the tested non-quartz sands are within the limits of this parameter given in the subject literature and indicate that they are alkaline, which excludes several organic binders, especially those hardened with acids.

The results for the tested non-quartz sands in Table 5 show that magnesite, chromite, and olivine sands meet the relevant standards for foundry sands. The examined sample of magnesite sand, despite meeting the criteria specified in the Polish standard [27] in terms of its particle size distribution, was classified into the 3rd grade of sand due to the presence of a binder in an amount exceeding, as shown in Table 4, the limit for grade 2 sand, amounting to 0.5%. Based on sieve analysis, chromite sand was assigned to the grain grade corresponding to coarse sand. However, due to the high binder content, the sand obtained the 3K grade according to the Polish Standard PN-91/H-11007 [27]. The results of the grain size distribution measurements for the P7-olivine OSF sand allowed it to be classified into the “fine” grain group and the 1K grade [28].

#### 3.2.2. XRD

**Figure 12:**
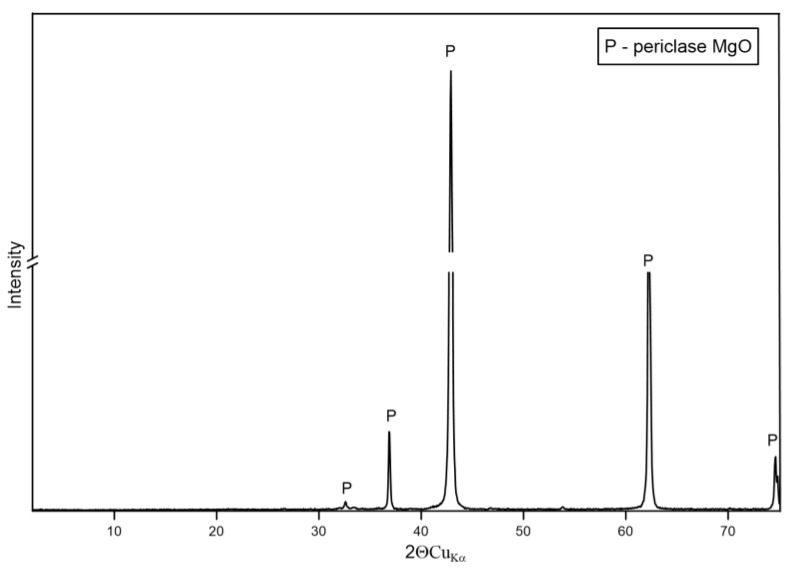
XRD pattern of sample P-4.

The XRD patterns for the non-silica sands, specifically magnesite (P-4), chromite (P-5), and olivine (P-7), reveal their mineral composition. The XRD analysis shows (Figure 12) that the dominant phase in magnesite sand is magnesium oxide (MgO), commonly referred to as periclase. The diffractogram exhibits characteristic peaks of periclase, confirming the purity of the sample. The crystallinity of the magnesite sand is high, as indicated by the sharpness and intensity of the peaks.

**Figure 13:**
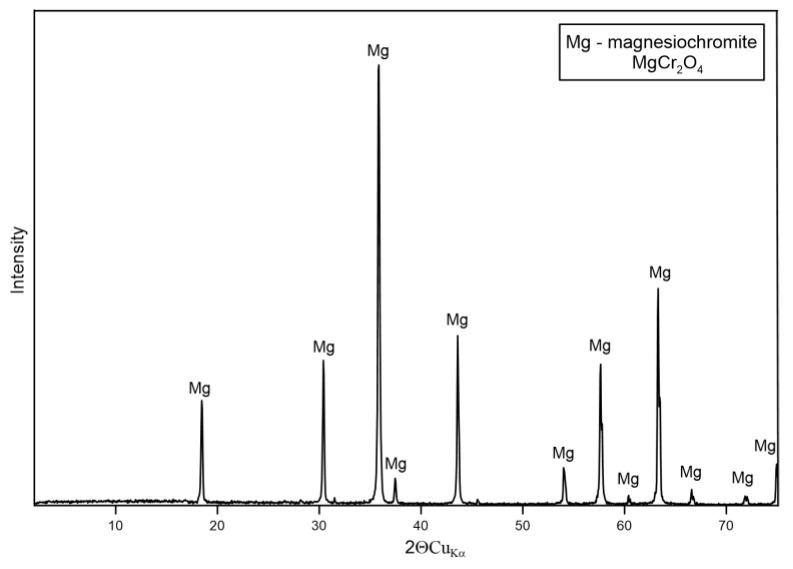
XRD pattern of sample P-5.

The chromite sand sample predominantly contains magnesiochromite (MgCr_2_O_4_) (Figure 13). The XRD pattern indicates that while magnesiochromite is the main mineral phase, there are slight peak shifts when compared to standard magnesiochromite, suggesting the presence of a mixed-phase structure, likely due to the incorporation of iron (Fe) and aluminum (Al) in the crystal lattice. Considering the content of Fe and Al in this sample, it should be assumed that it is not pure magnesiochromite but rather a phase with the composition (Mg,Fe)(Cr,Al)_2_O_4_. This is further supported by the SEM/EDS results, which show some amounts of Fe and Al within the magnesiochromite structure.

**Figure 14:**
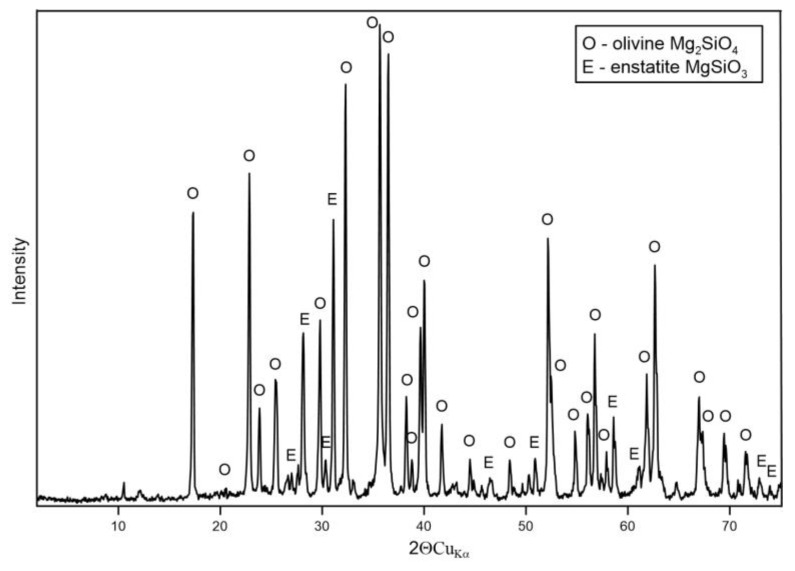
XRD pattern of sample P-7.

The XRD pattern for olivine sand shows (Figure 14) that the primary mineral phase is olivine (Mg_2_SiO_4_), a magnesium silicate commonly found in olivine-rich sands. In addition to olivine, minor peaks for enstatite (MgSiO_3_), another magnesium silicate, are also visible. Assuming that the percentages of SiO_2_ and MgO in the composition of olivine are 43.20% and 45.72%, respectively, the data in Table 5 indicate that the tested sample is forsterite. The peaks for forsterite are sharp, reflecting the high crystallinity of the sample, while the enstatite peaks are slightly less intense, indicating its presence in a smaller proportion. Both forsterite and enstatite suggest that the sand has undergone significant geological transformations, typical for olivine sands used in foundry applications.

In summary, the XRD results confirm that the non-silica sands are composed of mineral phases suitable for high-temperature foundry applications. Magnesite sand is primarily composed of MgO chromite sand, which is dominated by magnesiochromite with Fe and Al impurities, and olivine sand contains forsterite with minor enstatite. These mineralogical compositions provide the sands with unique refractory properties, making them suitable for casting applications with high thermal stability.

#### 3.2.3. SEM-EDS

The SEM and EDS analyses provided valuable insights into the microstructure and elemental composition of the non-silica sands, including magnesite (P-4), chromite (P-5), and olivine (P-7).

**Figure 15:**
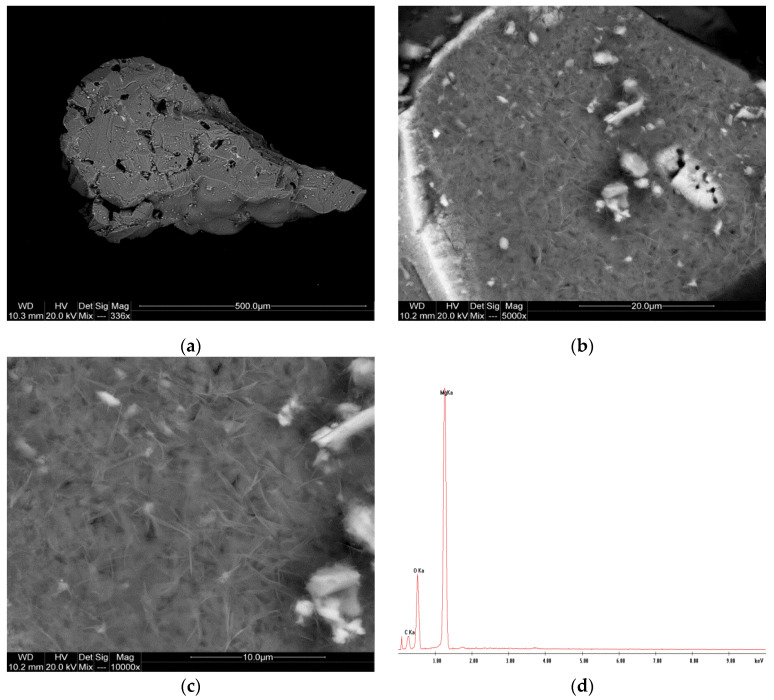
SEM images (**a**–**c**) and EDS spectrum (**d**) of the P-4 sample.

The SEM images of the magnesite sand (Figure 15) show a granular and relatively rough surface texture. Individual grains of the sample are angular and exhibit some minor porosity, which could impact its mechanical strength. The EDS spectrum confirms that the sand is predominantly composed of magnesium oxide (MgO), with slight traces of iron oxides (FeO), likely present as impurities or inclusions within the grains. These iron oxide inclusions, visible as bright spots in the SEM images, may influence the sand’s thermal and chemical stability under foundry conditions.

**Figure 16:**
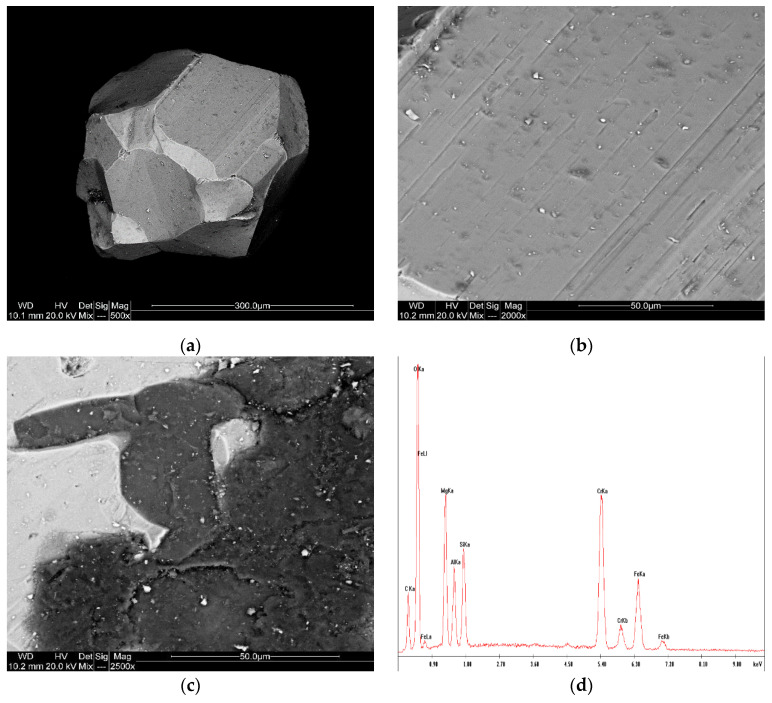
SEM images (**a**–**c**) and EDS spectrum (**d**) of the P-5 sample.

The chromite sand sample shows a more compact and dense microstructure than magnesite. SEM images (Figure 16) reveal rounded and angular grains with smooth surfaces. The EDS analysis highlights a composition dominated by chromium oxide (Cr_2_O_3_) and iron oxide (FeO), with significant amounts of magnesium oxide (MgO) and aluminum oxide (Al_2_O_3_). These observations support the conclusion that the chromite sand is a magnesiochromite mixture, as confirmed by the XRD analysis. Fe and Al within the chromite structure are consistent with previous studies on chromite sands, which typically contain these elements as part of their spinel structure.

**Figure 17:**
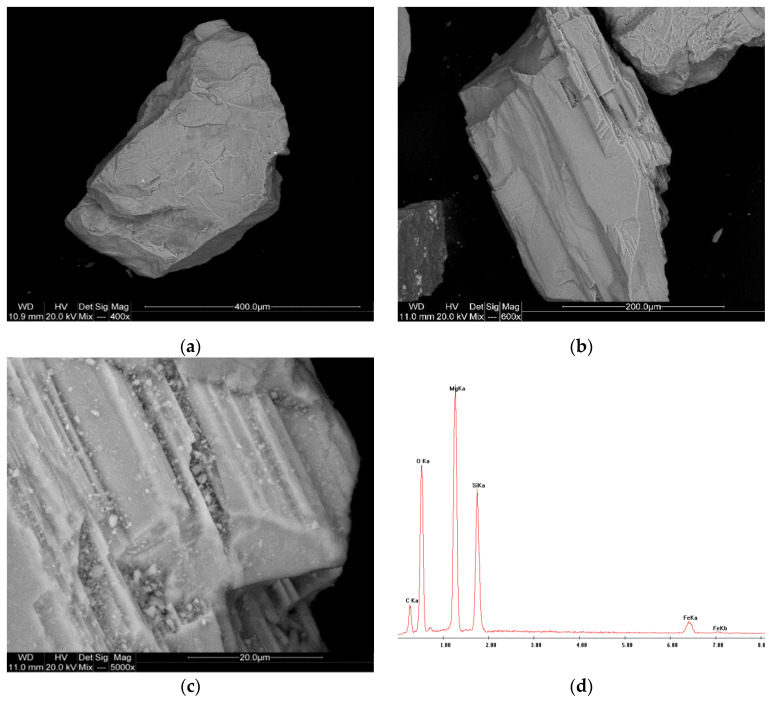
SEM images (**a**–**c**) and EDS spectrum (**d**) of the P-7 sample.

The SEM images of the olivine sand (Figure 17) show a more uniform grain distribution, with smooth, rounded particles and fewer irregularities. This morphology suggests that the sand has undergone significant transportation or weathering processes. The EDS spectrum identifies magnesium silicate (forsterite, Mg_2_SiO_4_) as the primary phase, with minor inclusions of enstatite (MgSiO_3_) and iron oxides. These inclusions are likely responsible for the observed secondary peaks in the XRD pattern. The high magnesium content (45.72%) and low silica content (43.20%) indicate that the sample is composed mainly of forsterite, making it suitable for high-temperature foundry applications due to its excellent refractory properties.

#### 3.2.4. TG/DTG

**Figure 18:**
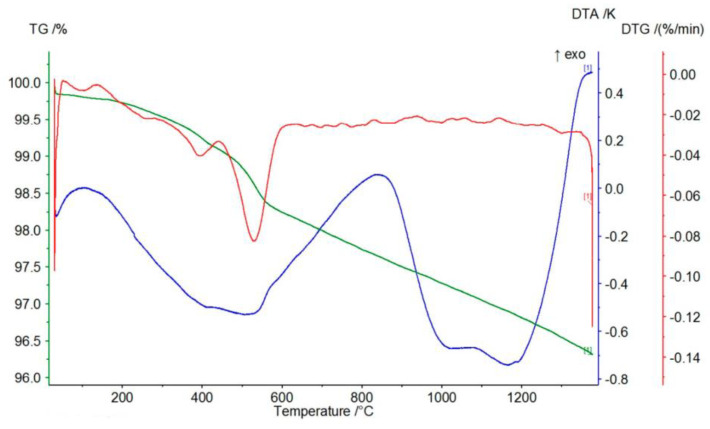
Thermal curves of sample P-4: TG curve (green), DTG curve (red), DTA curve (blue).

According to the course of the TG curve presented in Figure 18 and the data in Table 6, the tested sample is characterized by a monotonic, minimal mass loss of 3.42% at 1350 °C.

The presence of two endothermic peaks in the range of 400–500 °C in the DTG curve is reflected in the presence of a broad endothermic peak in the DTA curve with a minimum at a temperature of about 500 °C. In turn, the course of the DTG curve indicates that the observed mass loss is most likely related to the decomposition (dehydroxylation) of small amounts of magnesium oxide and calcium hydroxides (endothermic peaks on the TG curve at temperatures of 413 °C and 502 °C, respectively).

**Figure 19:**
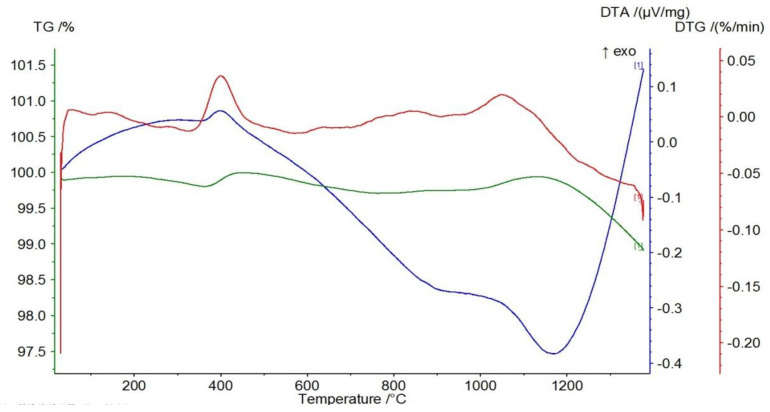
Thermal curves of sample P-5: TG curve (green), DTG curve (red), DTA curve (blue).

The course of the TG curve presented in Figure 19 and the data in Table 6 show that in the case of the chromite material, after evaporating the minimum amount of moisture (0.07%), heating the sample to about 450 °C causes an increase in mass at 447 °C by about 0.07%. This is reflected in the DTG and DTA curves by the appearance of exothermic peaks at temperatures around 378 and 387 °C, respectively. Further heating of the tested P-5 sample causes a mass decrease of about 0.16% at 1000 °C, further increasing by about 0.02% at a temperature of 1124 °C. This slight increase in mass is reflected in the appearance of two endothermic peaks on the DTG and DTA curves at the following temperatures: 820–890 °C and about 1100 °C.

The appearance of an exothermic peak at about 400 °C on both the DTG and DTA curves should be related to the oxidation of divalent to trivalent iron. During its heating, it diffuses from the inside of the chromite grains to their surfaces, creating a metastable spinel with a cation (FeOX_y_Fe_2_O_3_), ferrous–ferric oxide, which is oxidized to hematite at this temperature. Further heating causes the transformation of hematite to magnetite at a temperature of about 1100 °C [17]. The total mass loss of the P5 chromite sand at 1350 °C is about 0.82%.

**Figure 20:**
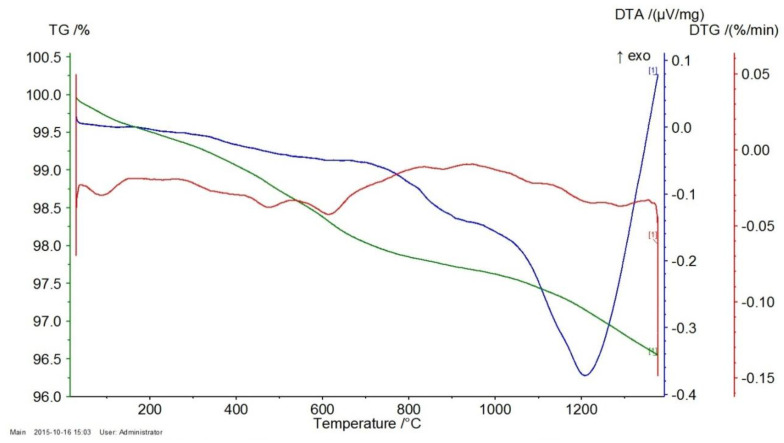
Thermal curves of sample P-7: TG curve (green), DTG curve (red), DTA curve (blue).

Figure 20 shows the course of the TG/DTG/DTA curves for the P-7 sample, and Table 6 presents the corresponding data obtained from the curves: the mass losses (%) that accompany the process of heating to the temperature of 1350 °C. The graphs of the TG/DTG/DTA curves show that the tested olivine sand is characterized by a practically monotonic mass loss during heating, which at 1350 °C equals 3.08%. Mass loss during heating is accompanied by practically one endothermic effect on the DTA curve with a minimum at the temperature of 1188 °C, showing the reaction of forsterite with amorphous enstatite, which leads to the formation of a crystalline form of the latter mineral, according to the following equation:

2Mg_2_[SiO_4_] + Mg_2_[Si_2_O_6_] -> 2Mg_2_[SiO_4_] + Mg_2_[Si_2_O_6_]

## 4. Discussion

This study sought to evaluate the physicochemical properties of various foundry sands—quartz, magnesite, chromite, and olivine—and determine their suitability for casting applications. It particularly focused on the critical processes of spheroidization and desulfurization in reactor housing production. These processes demand materials with precise thermal stability, chemical resistance, and structural integrity to ensure high-quality castings. Through a detailed investigation of grain size distribution, chemical composition, and thermal behavior, this study has elucidated key factors that influence the performance of these sands in foundry applications.

Quartz sands exhibit notable grain size distribution and chemical composition differences, even within the same classification. Sand P-12, with its uniform grain size distribution, demonstrated superior suitability for precise molding applications compared to P-11, which showed a broader range of finer particles. This inconsistency in particle size increases binder consumption and raises the risk of dimensional instability during high-temperature processes. Furthermore, thermogravimetric analysis revealed significant weight losses in P-11, linked to carbonates and natural binders. Such properties contribute to thermal instability, exacerbated by the polymorphic transformation of α-quartz to β-quartz, a well-known limitation of quartz sands. This transformation induces volumetric changes that can compromise mold integrity, leading to defects such as cracks and surface scabs in castings.

Magnesite sands, characterized by their high magnesium oxide (MgO) content, offer favorable thermal properties crucial for desulfurization and spheroidization. However, this study uncovered practical challenges related to storage and handling. Excessive loss on ignition, attributed to moisture absorption and oxidation during storage, suggests the need for stricter controls in material handling. While magnesite sands are suitable for high-temperature applications, these limitations could impair their performance in real-world scenarios, necessitating further refinement in storage and processing practices.

Chromite sands, renowned for their refractoriness and chemical stability, were evaluated for their mineralogical composition and thermal behavior. The presence of magnesiochromite, as confirmed by X-ray diffraction analysis, indicated a high degree of suitability for foundry use. However, impurities such as iron and aluminum oxides were also detected. These impurities can alter the sand’s thermal behavior and compromise its ability to resist slag formation, critical in preventing surface inclusions and ensuring smooth casting surfaces. Despite these impurities, chromite sands’ uniform thermal expansion properties remain a distinct advantage, making them highly desirable for specific casting applications.

Olivine sands, primarily composed of forsterite, demonstrated excellent thermal stability and mechanical strength, underscoring their potential for high-temperature foundry applications. However, deviations in composition, including slightly lower magnesium content and elevated iron content, posed challenges. High Fe_2_O_3_ levels increase slag formation risks, adversely affecting the mechanical properties and surface quality of castings. These findings suggest that while olivine sands are thermally robust, their compositional inconsistencies must be addressed to optimize their performance in precision casting.

This study effectively linked these findings to evaluating the compatibility of molding sands with specific casting processes. Grain size distribution emerged as a critical factor influencing mold stability and defect rates, with sands like P-12 showcasing the importance of uniform particle size. Similarly, the chemical purity of the sands was shown to directly impact their thermal and mechanical behavior. For example, high MgO levels in magnesite and olivine sands enhance their suitability for spheroidization and desulfurization, whereas excessive Fe_2_O_3_ content in chromite and olivine sands compromises their reliability.

These results have significant implications for foundry applications. Rigorous quality control and proactive testing of molding sands can prevent costly defects and enhance operational efficiency. Rejecting non-compliant sands early in production minimizes waste and ensures higher-quality castings. The findings also underscore the need for targeted material selection tailored to specific casting requirements, such as using magnesite sands for processes requiring high MgO content and chromite sands for applications demanding low thermal expansion.

The insights gained from this study pave the way for future research and innovation. Exploring advanced treatment methods to remove impurities, optimize grain size, and enhance thermal stability could significantly improve the performance of these materials. Additionally, the development of hybrid sands combining the strengths of different materials offers a promising avenue for meeting diverse industrial needs. Advanced monitoring systems that track the properties of sands in real time during production could also play a crucial role in ensuring consistency and quality.

## 5. Conclusions

This study evaluated the physicochemical properties of quartz, magnesite, chromite, and olivine sands to determine their suitability for reactor housing casting, focusing on spheroidization and desulfurization processes. The results underscore the importance of aligning molding sand properties with the stringent requirements of high-temperature casting applications.

Quartz sands exhibited significant variability in grain size distribution and thermal behavior, with P-12 showing promising uniformity and thermal stability. However, the broader particle size range and elevated binder content in P-11 highlight the need for quality control to prevent dimensional instability during casting. Magnesite sands demonstrated favorable properties due to their high MgO content, which supports desulfurization, but challenges with loss on ignition suggest improvements in storage and handling practices. Chromite sands, with their low thermal expansion and slag resistance, showed potential for high-temperature applications, although impurities such as iron and aluminum oxides warrant further refinement. Olivine sands, dominated by forsterite, exhibited excellent thermal properties but require adjustments to address deviations in MgO and Fe_2_O_3_ content for precision applications.

The findings of this study contribute to understanding how physicochemical properties impact the performance of molding sands in casting processes. This research provides actionable insights for optimizing sand selection and improving casting outcomes by systematically linking these properties to their industrial implications. Future work could focus on developing hybrid sands or advanced treatment techniques to address the limitations identified, ensuring consistent quality and performance in high-precision applications.

## Figures and Tables

**Table 1:** Statistical data on the particle size distribution of silica sands.

Silica Sand Parameters	P-3	P-11	P-12
Sand grain group	Medium	Fine	Coarse
Homogeneity index (%)	88.01	76.59	82.84
Average grain size (μm)	60	230	270
Average humidity (%)	0.2	0.02	0.08
Binder content (%)	1.9	0.16	0.11
Grade acc. PN-85/H-11001 [27]	4K	2K	1K

**Table 2:** List of selected parameters for quartz sands.

Parameter	P-3	P-11	P-12
pH	7.07	7.08	7.10
SiO_2_ (%)	99.40	98.88	96.81
Al_2_O_3_ (%)	0.21	0.81	1.44
Fe_2_O_3_ (%)	0.03	0.10	0.16
Carbonates (%)	0.23	0.15	0.17
LOI (%)	0.30	0.21	0.37

**Table 3:** Mass losses during heating of the P-3, P-11, and P-12 samples, based on the TG curve.

Temperature Range [°C]	P-3	P-11	P-12
	Mass Losses [%]		
25–100	0.16	0.05	0.03
100–600	0.90	0.57	0.66
100–1000	1.79	1.18	1.32
100–1350	2.44	1.64	1.81
750–850	0.23	0.15	0.17

**Table 4:** Statistical data on the particle size distribution of non-silica sands.

Non-Silica Sand Parameters	P-4 Magnesite	P-5 Chromite	P-7 Olivine
Sand grain group	Coarse	Coarse	Fine
Homogeneity index (%)	93.74	90.36	97.06
Average grain size (μm)	320	300	240
Average humidity (%)	0.07	0.03	0.04
Binder content (%)	0.83	0.80	0.46
Grade acc. PN-85/H-11001 [27]	3K	3K	1K

**Table 5:** List of selected parameters for non-silica sands.

Parameter	P-4	P-5	P-7
pH	12.25	10.21	10.52
Humidity (%)	0.07	0.03	0.04
SiO_2_ (%)	0.36	0.65	43.20
Al_2_O_3_ (%)	0.15	13.79	0.90
Fe_2_O_3_ (%)	0.44	23.47	8.55
Cr_2_O_3_ (%)	0.02	55.47	0.63
MgO (%)	98.17	6.83	45.72
CaO (%)	0.88	0.04	1.24
LOI (%)	1.20	1.96	0.19

**Table 6:** Mass losses during heating of the P-4, P-5, and P-7 samples, based on the TG curve.

Temperature Range [°C]	P-4	P-5	P-7
	Mass Losses [%]		
25–100	0.19	0.07	0.28
100–1000	2.53	0.16	3.06
1000–1350	3.42	0.82	3.08

## Data Availability

The original contributions presented in the study are included in the article, further inquiries can be directed to the corresponding author.
